# Biofilm-forming antimicrobial-resistant pathogenic *Escherichia coli*: A one health challenge in Northeast India

**DOI:** 10.1016/j.heliyon.2023.e20059

**Published:** 2023-09-12

**Authors:** A. Arun Prince Milton, K. Srinivas, Vanita Lyngdoh, Aleimo G. Momin, Naphisabet Lapang, G. Bhuvana Priya, Sandeep Ghatak, R.K. Sanjukta, Arnab Sen, Samir Das

**Affiliations:** a Division of Animal and Fisheries Sciences, ICAR Research Complex for Northeastern Hill Region, Umiam, Meghalaya, India; b College of Agriculture, Central Agricultural University (Imphal), Kyrdemkulai, Meghalaya, India

**Keywords:** *E. coli*, STEC, EPEC, Biofilm, ESBL, PFGE

## Abstract

This study aimed to investigate the prevalence of Shiga toxin-producing *Escherichia coli* (STEC), Enteropathogenic *E. coli* (EPEC), and Enterotoxigenic *E. coli* (ETEC) in common food animals (cattle, goats, and pigs) reared by tribal communities and smallholder farmers in Northeast India. The isolates were characterized for the presence of virulence genes, extended-spectrum beta-lactamases (ESBL) production, antimicrobial resistance, and biofilm production, and the results were statistically interpreted. In pathotyping 141 *E. coli* isolates, 10 (7.09%, 95% CI: 3.45%–12.66%) were identified as STEC, 2 (1.42%, 95% CI: 0.17%–5.03%) as atypical-EPEC, and 1 (0.71%, 95% CI: 0.02%–3.89%) as typical-EPEC. None of the isolates were classified as ETEC. Additionally, using the phenotypic combination disc method (ceftazidime with and without clavulanic acid), six isolates (46.1%, 95% CI: 19.22%–74.87%) were determined to be ESBL producers. Among the STEC/EPEC strains, eleven (84.6%, 95% CI: 54.55%–98.08%) and one (7.7%, 95% CI: 0.19%–36.03%) strains were capable of producing strong or moderate biofilms, respectively. PFGE analysis revealed indistinguishable patterns for certain isolates, suggesting clonal relationships. These findings highlight the potential role of food animals reared by tribal communities and smallholder farmers as reservoirs of virulent biofilm-forming *E. coli* pathotypes, with implications for food contamination and zoonotic infections. Therefore, monitoring these pathogens in food animals is crucial for optimizing public health through one health strategy.

## Graphical abstract

**Figure:**
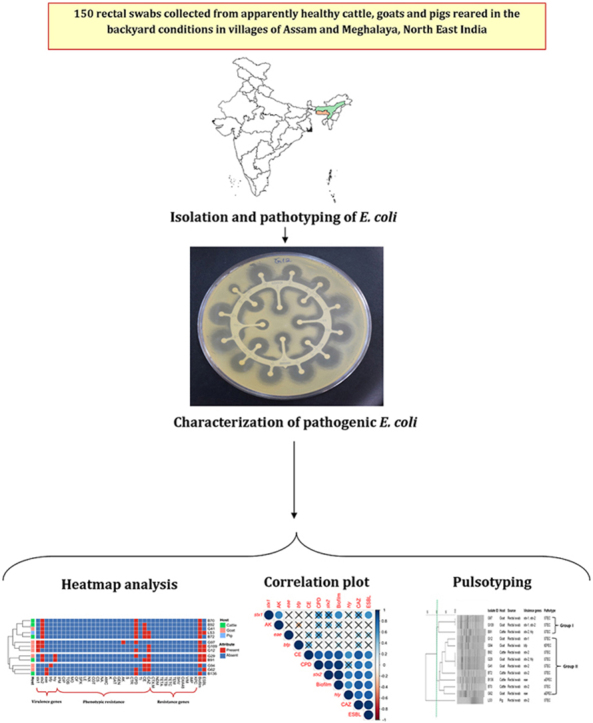

## 1 Introduction

*Escherichia coli* (*E. coli*) is a Gram-negative facultative anaerobic bacterium that is an innocuous occupant of the intestinal tract of warm-blooded animals and humans. However, *E. coli* has the potential to cause severe gastrointestinal (diarrhoeal) and extraintestinal diseases [1]. *E. coli* strains incriminated in diarrhoeal cases are jointly referred to as diarrheagenic *E. coli* (DEC), a collection comprising enterohemorrhagic *E. coli* (EHEC), shigatoxigenic *E. coli* (STEC), enteropathogenic *E. coli* (EPEC), enterotoxigenic *E. coli* (ETEC), enteroaggregative *E. coli* (EAEC), diffuse-adherent *E. coli* (DAEC), and enteroinvasive *E. coli* (EIEC). Among these strains, STEC/EHEC and EPEC constitute a significant zoonotic concern, which was established in multiple earlier studies [1,2]. STEC colonises the large intestine via an attaching and effacing lesion. This lesion is caused by a bacterial type III secretion system that injects effector proteins into the intestinal epithelial cell, resulting in profound alterations to the host cell's architecture and metabolism and the close adherence of the bacteria. Shiga toxin (Stx), which exists in two main forms, Stx1 and Stx2, is responsible for severe diseases such as bloody diarrhoea and the hemolytic uremic syndrome. The *stx* genes are encoded on the chromosomes of temperate bacteriophages, and production and release of the toxin are highly dependent on phage induction [3]. Ruminants, being the major animal reservoirs of STEC, are usually asymptomatic when colonized; however, a few STEC strains may cause diarrhoea in calves [4].

EPEC strains, classified as typical EPEC (tEPEC) and atypical EPEC (aEPEC), are responsible for causing fatal diarrhoea characterized by the presence of attaching and effacing (A/E) lesions in the intestine. These lesions are mediated by intimin, which is encoded by the *eae* gene. Typical EPEC strains carry an EPEC adherence factor (EAF) plasmid containing the bfp operon, which encodes the bundle-forming pilus (BFP), while atypical EPEC strains lack the EAF plasmid [5]. The central mechanism behind EPEC pathogenesis is the formation of A/E lesions, which involve the destruction of microvilli, intimate adherence of bacteria to the intestinal epithelium, pedestal formation, and aggregation of polarized actin and other cytoskeletal elements at sites of bacterial attachment. The genetic determinants responsible for the production of these A/E lesions are located on the locus of enterocyte effacement (LEE), which is a pathogenicity island containing genes encoding intimin, a type III secretion system, various secreted (Esp) proteins, and the translocated intimin receptor named Tir [5]. Humans are the main natural reservoir of tEPEC, and they have been rarely isolated from animals. However, aEPEC strains have been repeatedly isolated from both diseased and healthy animals and humans [2]. Several studies have demonstrated that STEC and EPEC strains from humans and animals share virulence characteristics and are clonally related [6].

The emergence of multi-drug resistance (MDR) in *E. coli* strains is complicating the treatment of many serious infections and is a serious public health concern. Earlier studies consistently reported an increase in antimicrobial resistance (AMR) among pathogenic *E. coli* strains isolated from both human and animal clinical samples [7]. Particularly, the emergence of extended-spectrum beta-lactamases (ESBL) production and carbapenem resistance in *E. coli* strains is a serious threat to human health. The development of antimicrobial resistance in *E. coli* is usually due to the plasmid-mediated acquisition of antimicrobial resistant genes (ARGs). *E. coli* employs a variety of strategies to survive and persist in the environment, and several studies have documented the biofilm-forming capabilities of different *E. coli* pathotypes [8,9]. The biofilm formation followed by the encasement of *E. coli* in a complex matrix can augment resistance to antibiotics and sanitising agents, leading to difficulty in eradicating and controlling them [9]. Since MDR *E. coli* strains are becoming common and can form biofilms, it is essential that new, effective, and safe antimicrobial drugs be developed. These drugs should combat the difficulties of biofilm formation while being effective against resistant bacteria, such as those that produce ESBL and display carbapenem resistance.

The prevalence of diarrhoea, especially in below-five-year-old children is 10.6% and 2.5% in Meghalaya and Assam (Northeastern states of India), respectively [10]. But the prevalence of STEC, EPEC, and ETEC in food animals and their virulence potential are not well studied, and there is a paucity of studies. In this study, we examined rectal swabs obtained from commonly raised food animals (cattle, goat, and pig) in Northeastern India's tribal communities and smallholder farms located in Meghalaya and Assam. Our primary objective was to assess the prevalence of *E. coli* pathotypes (STEC, EPEC, and ETEC) and investigate potential correlations among virulence genes, phenotypic antimicrobial resistance, ARGs, biofilm-forming ability, and PFGE fingerprinting within these specific *E. coli* pathotypes.

## 2 Materials and methods

### 2.1 Sample collection

**Fig. 1:**
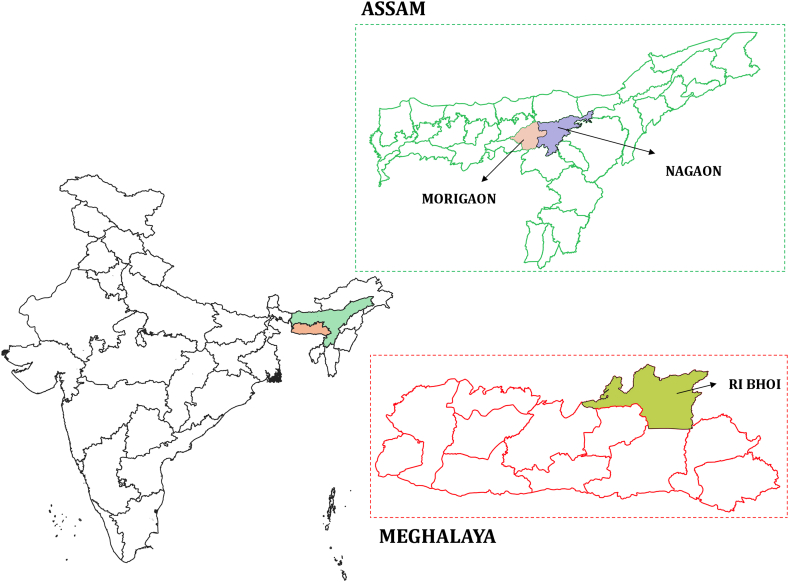
Map depicting the study area in Northeastern India, highlighting the districts where samples were collected from Assam and Meghalaya.

During 2019–2020, a total of 150 rectal swabs were collected from apparently healthy cattle (n = 50), goats (n = 50), and pigs (n = 50) reared in backyard conditions in villages in Meghalaya (Ri Bhoi District) and Assam (Nagaon and Morigaon Districts) of Northeastern India (Fig. 1). All of these animals were raised in a semi-intensive housing system following conventional management practices. It is important to note that the animals received minimal or no antibiotic treatment, and specifically, antibiotics in the form of growth promoters were not administered to them. The studied households only had 1-2 animals per household, and feeding was usually managed with kitchen waste, locally available fodder, raw cereals, and vegetables. As the majority of pigs are raised in traditional small-holder systems, convenient sampling was conducted by obtaining one sample from each randomly chosen household or farm. The ethical approval for conducting the study with non-invasive sampling was obtained from the Institutional Animal Ethics Committee (IAEC) of ICAR RC NEH, Meghalaya, which is regulated by the Committee for Control and Supervision of Experiments on Animals (CCSEA) guidelines. The rectal swabs were aseptically collected with the help of a sample collector fixed with a cotton swab and sterile transport medium (HiMedia, Mumbai, India). The swabs, after soaking in the transport medium, were gently inserted into the rectum of the animals to collect the samples. The collected samples were transported to the laboratory under chilled conditions and immediately processed for the isolation of *E. coli.*

### 2.2 Isolation and identification of *E. coli* and PCR-based confirmation

The isolation and identification of *E. coli* were done as previously described (Milton et al., 2019). The collected samples were inoculated into MacConkey broth (HiMedia, India) and incubated at 37 °C for 24 h. After enrichment, the inoculum was streaked onto eosin methylene blue (EMB) agar (HiMedia, India) and incubated at 37 °C for 24 h. The colonies with a green metallic sheen were further streaked into nutrient agar slants (HiMedia, India) for further biochemical characterization (indole production, methyl red test, Voges-Proskauer test, and citrate utilisation test) and PCR confirmation. The isolates were grown in Luria Bertani broth (HiMedia, India), and genomic DNA was extracted employing the QIAamp DNA Mini Kit (Qiagen, Hilden, Germany). PCR analysis using co-amplification of five genes, i.e., *lacY* [11], *lacZ* [12], *cyd* [11], *phoA* [13], and *uidA* [12], was done for specific confirmation of *E. coli* and differentiation from *Shigella* spp. and other species of *Enterobacteriaceae*. *E. coli* ATCC 25922 was used as the reference strain.

### 2.3 Pathotyping and detection of other virulence genes

For pathotyping the recovered *E. coli* isolates into STEC, EPEC, and ETEC, PCR screening of *stx1* [14], *stx2* [14], *eae* [15], *bfp* [15], *stII* [15], and *lt* [15] genes were done using previously described methods. Further, the presence of serotype-specific virulence genes such as *wzx*_O104_, *fli*C_H4_, *rfb*_O157_, and *fli*C_H7_ [16] was also determined using PCR-based analysis. The presence of the *hlyA* gene was also detected using a previously described PCR protocol [17]. The reference strains and field isolates from our previous studies were used as positive controls.

### 2.4 Phenotypic analysis of ESBL production and antimicrobial resistance

Only the pathotypes recovered from the study were included in the analysis of antimicrobial resistance. ESBL production was tested phenotypically by employing the combined disc method using ceftazidime (CTZ, 30 μg) with and without clavulanic acid (30 μg/10 μg) and cefotaxime (CTX, 30 μg) with and without clavulanic acid (30 μg/10 μg). The isolates were classified as ESBL producers if the inhibition between the single and combination antibiotic discs differed by ≥ 5 mm. The pathogenic *E. coli* isolates recovered were subjected to phenotypic testing of antibiotic susceptibility using 20 commercial antibiotic discs (HiMedia). Antimicrobial susceptibility testing was performed on Mueller Hinton agar as per the protocols of the Clinical Laboratory Standard Institute [18]. The antibiotic discs used in the study belong to various antimicrobial classes. These include tetracycline (TE, 30 μg) from the tetracyclines class, imipenem (IPM, 10 μg) from the carbapenems class, tobramycin (TOB, 10 μg) from the aminoglycosides class, ciprofloxacin (CIP, 5 μg), moxifloxacin (MXF, 5 μg), ofloxacin (OFX, 5 μg), sparfloxacin (SPX, 5 μg), levofloxacin (LVX, 5 μg), and norfloxacin (NOR, 10 μg) from the fluoroquinolones class. Other antibiotic discs include co-trimoxazole (COT, 25 μg) from the sulfonamides/trimethoprim class, colistin (CST, 10 μg) from the polymyxins class, nalidixic acid (NAL, 30 μg) from the quinolones class, augmentin (AMC, 30 μg) from the penicillin/beta-lactamase inhibitor combination class, kanamycin (KAN, 30 μg), gatifloxacin (GAT, 5 μg), gentamicin (GEN, 10 μg), amikacin (AN, 30 μg), and streptomycin (STR, 10 μg) from the aminoglycosides class. Cefpodoxime (CPD, 10 μg) belonging to the cephalosporin class, and ticarcillin (TIC, 75 μg) belonging to the penicillin class were also used. The interpretation for antibiotic susceptibility testing (sensitive, intermediate, or resistant) was done following the chart supplied by the manufacturer based on the recommendations of CLSI and EUCAST.

### 2.5 Detection of antimicrobial resistance genes

The genomic DNA extracted from recovered *E. coli* pathotypes was subjected to PCR analysis for the detection of frequent AMR genes such as *bla*_TEM_ [19], *bla*_NDM_ [20]*, bla*_CTX-M_ [21], *bla*_IMP_ [22], *bla*_VIM_ [22], *bla*_SHV_ [23], *bla*_KPC_ [22], *tetA* [24]*, tetB* [24]*, tetC* [25], and *OXA-48* [23]. The reference strains and field isolates from our previous studies were used as positive controls.

### 2.6 Analysis of biofilm production

Biofilm formation analysis was performed following a previously described microtiter plate-based method [26]. The recovered pathogenic *E. coli* isolates were grown in LB broth (5 mL) at 35.5 °C overnight. The grown cultures (1.3 μL) were added to 130 μL of M9 broth with 0.8% glucose in polystyrene microtitre plates (96 wells/flat bottom) and incubated at 30 °C overnight, and the optical density (OD) of the wells was measured (λ = 620 nm). Then the broth was discarded, and wells were rinsed with sterile saline (150 μL) to eliminate nonadherent bacteria. After air drying for 20 min, 1% crystal violet solution (130 μL/well) was added and kept for 5 min. Later on, the colourant was removed, and the wells were washed three times with distilled water (180 μL) to remove excess stain. After airdrying for 1 h, 130 μL of absolute ethanol was added to solubilize the dye incorporated by the adhered bacterial cells. Finally, the OD of the test and control wells was read at λ = 540 nm. *E. coli* ATCC 25922 was used as a control in the experiment. The degree of biofilm formation was determined by applying a specific biofilm formation (SBF) index formula, SBF = (AB − CW) G, where AB is the OD_540__nm_ of stained adhered cells; CW is the OD_540__nm_ of stained control wells containing only M9 medium; and G is the OD_620__nm_ of growth in suspended culture. The isolates were classified as strong (≥1.10), moderate (0.70–1.09), weak (0.35–0.69), and negative (<0.35) biofilm producers, as described by Naves et al. [26].

### 2.7 PFGE fingerprinting and analysis

Pulsed-field gel electrophoresis (PFGE) was performed only on pathogenic *E. coli* strains (n = 13) as per the Centers for Disease Control and Prevention (CDC) guidelines using the PulseNet (24–26 h) standardized protocol. *Xba*I (50 U) restriction endonuclease (Thermo Scientific) was used to digest the DNA for each isolate, and CHEF DNA Size Marker- *S. cerevisiae* (BIO-RAD Labs, Hercules, CA) was used as a molecular size marker ranging from 225 kb to 2200 kb. The digested DNA was electrophoresed in the CHEF-DR® III System (BIO-RAD) with the following conditions: temperature of 14 °C; 120° switch angle; 6 V/cm voltage; initial and final switch times of 6.76 s and 35.38 s, respectively; and a 20 h run time. The fingerprints were analyzed with Bionumerics v7.6 fingerprinting software (Applied Maths, Belgium). To check the genetic relatedness of the isolates based on the number of bands produced, cluster analysis employing the unweighted pair group method with averaging (UPGMA) was done to construct a dendrogram, unfolding the relationship among PFGE patterns (% similarity). The pathogenic *E. coli* strains were fitted to the same PFGE cluster if their index of similarity was ≥90%.

### 2.8 Statistical analysis

Statistical association between variables such as pathotypes, biofilm formation, and ESBL production was studied. The data was analyzed using Fisher's exact test to test hypotheses, with p-values calculated using MS-Excel. A heatmap was constructed using the “pheatmap” package with hierarchical clustering on R software (4.2.2 version). A contingency table of the attributes was prepared based on which a correlation plot with Spearman's rank correlation methods was generated using the “corrplot” package of R software (4.2.2 version).

## 3 Results

### 3.1 Isolation of *E. coli* and prevalent *E. coli* pathotypes

In this study, 141 (141/150, 94%, 95% CI: 88.92%–97.22%) isolates were isolated and confirmed as *E. coli* using morphological and biochemical characterization and PCR analysis. Based on PCR pathotyping, 10 (10/141; 7.09%, 95% CI: 3.45%–12.66%), 2 (2/141; 1.42%, 95% CI: 0.17%–5.03%), and 1 (0.71%, 95% CI: 0.02%–3.89%) *E. coli* isolates were classified as STEC, atypical EPEC, and typical EPEC, respectively, and none of the isolates were typed as ETEC. The distribution of various virulence genes in the isolates is presented in Table 1. Out of 10 STEC isolates, 5 (5/50; 10%, 95% CI: 3.33%–21.81%), 4 (4/50; 8%, 95% CI: 2.22%–19.23%), and 1 (1/50; 2%, 95% CI: 0.05%–10.65%) were isolated from rectal swabs of goats, cattle, and pigs, respectively. EPEC was isolated from two goats (2/50; 4%, 95% CI: 0.49%–13.71%) and one cattle (1/50; 2%, 95% CI: 0.05%–10.65%). One EPEC was isolated from goats (1/50; 2%, 95% CI: 0.05%–10.65%) and cattle (1/50; 2%, 95% CI: 0.05%–10.65%), and a tEPEC was isolated from a goat (1/50; 2%, 95% CI: 0.05%–10.65%). The *hlyA* gene was detected in two STEC isolates from goats and cattle. None of the *E. coli* pathotypes were found to harbour the other targeted genes, i.e., *wzx*_O104_ and *fli*C_H4_, *rfb*_O157_, and *fli*C_H7._

**Table 1:** Complete characterization profile of the pathogenic *E. coli* isolated from food animals.

Sample ID.	Host	Virulence	*E. coli* Pathotype	ESBL Production (Phenotypic)	Biofilm producing ability	Antimicrobial-resistant pattern
G62	Goat	*eae*	aEPEC	ESBL producer	Non-biofilm producer (−0.036)	CPD,CTZ,CTX
B136	Cattle	*eae*	aEPEC	Non-ESBL producer	Strong (2.064)	CPD
G94	Goat	*bfp*	tEPEC	Non-ESBL producer	Strong (1.718)	CTX,CTZ
B92	Cattle	*stx2*	STEC	Non-ESBL producer	Strong (1.637)	CPD,CTX
LS3	Pig	*stx2*	STEC	ESBL producer	Strong (1.345)	CPD,CTZ,CTX
G109	Goat	*stx1, stx2*	STEC	ESBL producer	Strong (1.109)	CTZ
B70	Cattle	*stx2*	STEC	Non-ESBL producer	Strong (1.865)	CPD
G12	Goat	*stx1*	STEC	ESBL producer	Strong (1.709)	CPD,CTZ
G97	Goat	*stx1, stx2*	STEC	Non-ESBL producer	Moderate (0.851)	AK,CPD
B72	Cattle	*stx2*	STEC	Non-ESBL producer	Strong (1.666)	CPD,CTZ,CTX
G41	Goat	*stx2*	STEC	Non-ESBL producer	Strong (2.486)	CPD,CTX
G29	Goat	*stx2. hly*	STEC	ESBL producer	Strong (1.827)	CPD,CTZ
B91	Cattle	*stx2, hly*	STEC	ESBL producer	Strong (2.964)	CTX,CTZ

### 3.2 Phenotypic characteristics of the recovered isolates

The antimicrobial resistance pattern of the isolated *E. coli* pathotypes (n = 13) is furnished in Table 2. The resistances to amikacin, cefpodoxime, cefotaxime, and ceftazidime were found to be 7.7% (95% CI: 0.19%–36.03%), 77% (95% CI: 46.19%–94.96%), 53.8% (95% CI: 25.13%–80.78%), and 61.5% (95% CI: 31.58%–86.14%), respectively. No resistance was found against tetracycline, imipenem, tobramycin, ciprofloxacin, moxifloxacin, ofloxacin, sparfloxacin, levofloxacin, norfloxacin, co-trimoxazole, colistin, nalidixic acid, augmentin, kanamycin, gatifloxacin, gentamicin, streptomycin, ceftriaxone, and ticarcillin. Based on the phenotypic combination disc (ceftazidime with and without clavulanic acid) method, 6 of the 13 isolates (6/13; 46.1%, 95% CI: 19.22%–74.87%; 5 STEC and 1 aEPEC) were found to be ESBL producers (Table 1). In the current study, however, none of the isolates were found to have any of the antimicrobial resistance genes that were tested: *blaTEM*, *blaNDM*, *blaSHV, blaCTX-M, bla*_IMP_, *bla*_KPC_, *bla*_VIM_, *tetA, tetB, tetC,* or *OXA-48.* None of the pathogenic *E. coli* isolates in the present study were multi-drug-resistant.

**Table 2:** Antimicrobial resistance pattern of pathogenic *E. coli* isolated from food animals.

Antimicrobial agents used	Sensitive	Intermediate	Resistant
Ceftazidime (CTZ, 30 μg)	1 (7.7%)	4 (30.7%)	8 (61.5%)
Ceftazidime/clavulanic acid (CEC; 30 μg/10 μg)	12 (92.3%)	1 (7.7%)	0
Cefotaxime (CTX, 30 μg)	1 (7.7%)	5 (38.5%)	7 (53.8%)
Cefotaxime/clavulanic acid (CAC; 30 μg/10 μg)	1 (7.7%)	1 (7.7%)	11 (84.6%)
Tetracycline (TE, 30 μg)	13 (100%)	0	0
Imipenem (IPM, 10 μg)	12 (92.3%)	1 (7.7%)	0
Tobramycin (TOB, 10 μg)	12 (92.3%)	1 (7.7%)	0
Ciprofloxacin (CIP, 5 μg)	13 (100%)	0	0
Moxifloxacin (MXF, 5 μg)	13 (100%)	0	0
Ofloxacin (OFX, 5 μg)	13 (100%)	0	0
Sparfloxacin (SPX, 5 μg)	13 (100%)	0	0
Levofloxacin (LVX, 5 μg)	13 (100%)	0	0
Norfloxacin (NOR, 10 μg)	13 (100%)	0	0
Co-trimoxazole (COT, 25 μg)	13 (100%)	0	0
Colistin (CST, 10 μg)	13 (100%)	0	0
Nalidixic acid (NAL, 30 μg)	13 (100%)	0	0
Augmentin (AMC, 30 μg),	13 (100%)	0	0
Kanamycin (KAN, 30 μg)	6 (46.1%)	7 (53.8%)	0
Gatifloxacin (GAT, 5 μg)	13 (100%)	0	0
Gentamicin (GEN, 10 μg)	13 (100%)	0	0
Amikacin (AN, 30 μg)	12 (92.3%)	0	1 (7.7%)
Streptomycin (STR, 10 μg)	12 (92.3%)	1 (7.7%)	0
Cefpodoxime (CPD, 10 μg)	0	3 (23%)	10 (76.9%)
Ticarcillin (TIC, 75 μg)	10 (76.9%)	3 (23%)	0

### 3.3 Biofilm-producing ability

Using a microtiter plate-based method and a specific biofilm formation (SBF) index formula, we found that 12 out of 13 recovered pathogenic *E. coli* isolates were able to form biofilm, with an average absorbance (total) of 1.668 (Table 1). Among the biofilm-producing strains, eleven and one strain formed strong and moderate biofilms, respectively. The average absorbance of the strong biofilm-producing isolate was 1.854 (ranging from 1.109 to 2.064), and the absorbance of the moderate biofilm-producing isolate was 0.851. Except for one non-ESBL producer (G62), all five (LS3, G109, G12, G29, and B91) were observed as strong biofilm producers.

### 3.4 PFGE fingerprinting

**Fig. 2:**
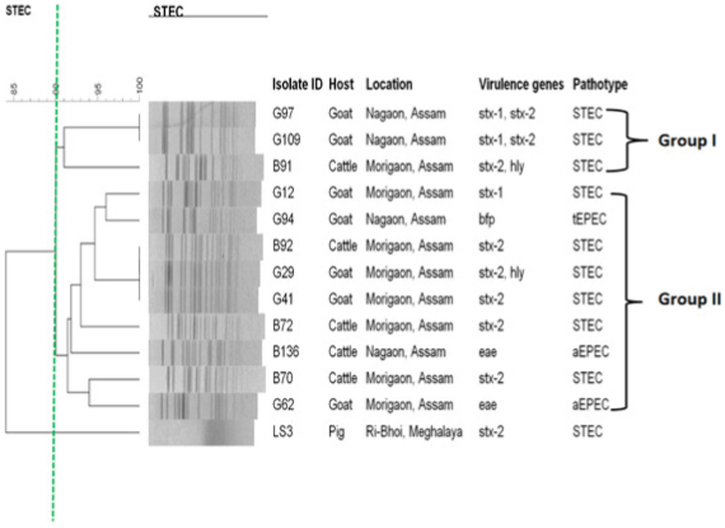
Dendrogram of PFGE fingerprinting of pathogenic *E. coli* isolates from food animals.

The genetic relatedness of *Xba*I-digested *E. coli* isolates was determined by analyzing their PFGE patterns. Out of the 13 pathogenic *E. coli* isolates digested with *Xba*I, 9 different PFGE patterns (pulsotypes) were identified. However, one isolate (LS3, an STEC from a pig) could not be typed. Interestingly, STEC isolates (*stx1*^+^ and *stx2*^+^) from goats (G97 and G109) showed PFGE patterns with 100% similarity, indicating a close genetic relationship. In addition, three STEC isolates from goats and cattle, namely G29 (*stx2*^+^, *hlyA*^+^), G41 (*stx2*^+^), and B92 (*stx2*^+^), exhibited indistinguishable PFGE patterns, despite their differences in biofilm-producing ability (G97-moderate biofilm and G109-strong biofilm) and antimicrobial resistance (B92-non-ESBL producer and G29-ESBL producer). The nine PFGE profiles were further classified into two groups (Group I and II) based on their 90% similarity, as depicted in the dendrogram shown in Fig. 2. This clustering implies that similar *E. coli* pathotypes tend to group together and share a clonal relationship. Overall, the findings suggest that similar pathotypes of *E. coli* (such as STEC) generate comparable PFGE patterns, regardless of their differences in biofilm formation or antimicrobial resistance.

### 3.5 Statistical analysis

**Fig. 3:**
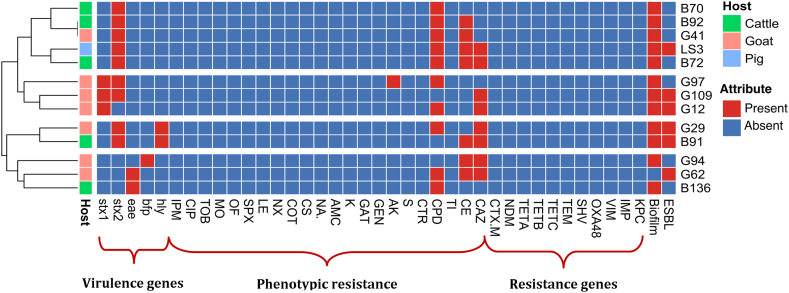
Heatmap analysis for host, pathotype, antimicrobial resistance, antimicrobial resistant genes and biofilm formation.

**Fig. 4:**
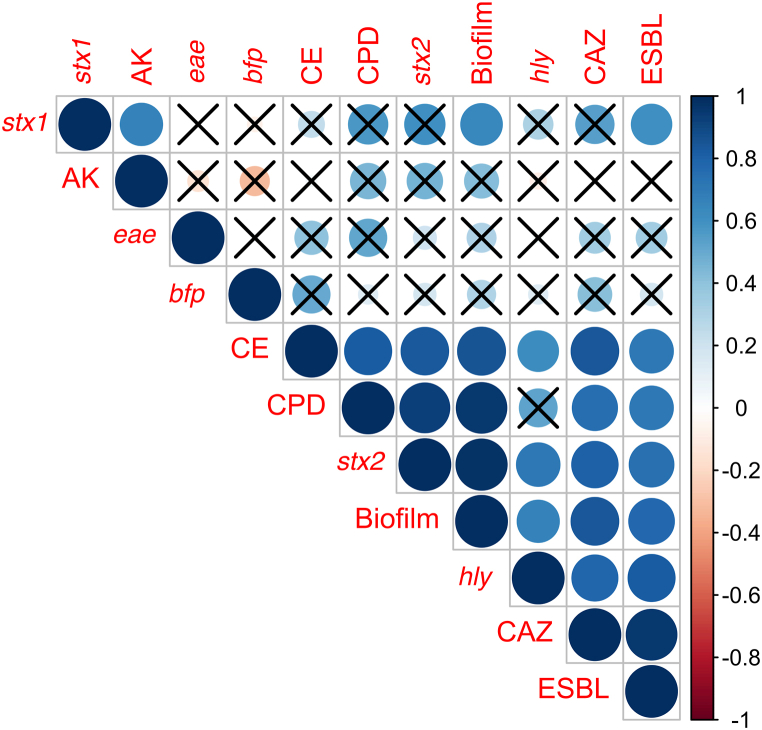
Correlation matrix for pathotype, antimicrobial resistance, ESBL production and biofilm formation.

Based on their phenotypic and genotypic traits, heatmap analysis showed that the isolates from the different hosts grouped together (Fig. 3). Furthermore, the correlation plot revealed significant positive associations among certain traits. Biofilm formation, cefpodoxime resistance, cefotaxime resistance, and the presence of the *stx2* gene showed a strong positive correlation (Spearman's coefficient [ρ] > 0.92, p < 0.05) (Fig. 4). Similarly, as expected, ceftazidime resistance exhibited positive correlations with ESBL production and cefotaxime resistance (ρ > 0.7, p < 0.05). Additionally, the presence of the *hlyA* gene showed a positive correlation with ESBL production (ρ = 0.82, p < 0.05).

## 4 Discussion

This study investigated the presence of STEC, EPEC, and ETEC in the faeces of cattle, goats, and pigs reared by tribal people and smallholder farmers in Northeast India. Isolates were characterised for virulence genes, ESBL production, antibiotic resistance, and biofilm formation. The recovered isolates of pathogenic *E. coli* were fingerprinted with PFGE. On the basis of PCR-based pathotyping, 10 *E. coli* isolates were classified as STEC (7.09%), 2 as atypical EPEC (1.42%), and 1 as typical EPEC (0.71%); none of the isolates were typed as ETEC. In concordance with our study, a recent study from Northeast India has also documented a prevalence of 9.88% and 4.32% of STEC and EPEC, respectively, in small ruminants and associated samples. However, the same study documented a 12% prevalence of ETEC, whereas no ETEC was isolated in our study [27]. Similar to the present study, a previous Indian study also described STEC followed by EPEC as the most prevalent pathotypes in neonatal calves [28]. A previous study from Spain has also described the high prevalence of STEC and EPEC in goat's milk [29]. In concordance with our study results, a lower rate of ETEC among food animals was previously described by many researchers around the world [[30], [31], [32]]. The above observations are significant from a public health and zoonotic perspective, as the faecal carriage of *E. coli* pathotypes can contaminate foods of animal origin and can also transmit to humans through direct contact.

We found that resistance to Amikacin was 7.7%, cefpodoxime was 77%, cefotaxime was 53.8%, and ceftazidime was 61.5%. Out of the isolates tested using the phenotypic combination disc method (ceftazidime with and without clavulanic acid), 46.1% (six isolates) showed positive results for ESBLs. The recovered pathogenic *E. coli* isolates did not harbour any of the tested antimicrobial resistance genes. It is quite evident from earlier observations that high antimicrobial resistance is usually associated with non-pathogenic or commensal strains [33,34]. However, in some other studies, virulent microbes have displayed high AMR patterns as well [35,36]. Further, in our study, the sampled animals were mostly reared by tribal communities and smallholder farmers in Northeastern India, who usually do not get much access to antibiotic treatment.

Twelve pathogenic *E. coli* isolates (92.3%) formed biofilm. The ability to form biofilms is crucial for any bacterial pathogen as it plays a significant role in infecting the host and contributing to its pathogenicity. For bacterial pathogens, biofilm formation facilitates long-term colonization and provides protection against antimicrobials and the host immune system [8]. In multiple studies, the pathotypes of *E. coli* have been demonstrated to be strong and moderate biofilm formers [37,38]. The results of a previous study have also indicated that the biofilm-forming ability could contribute to the persistence of STEC [37].

This study demonstrates that pathogenic *E. coli* strains of the same pathotype (STEC) exhibit indistinguishable PFGE patterns, regardless of their biofilm-forming capacity and antibiotic sensitivity. The PGFE analysis of these isolates establishes their identical nature. Previously, PFGE typing has been effectively used multiple times to decipher the genetic relatedness of *E. coli* pathotypes from various food animals [39,40]. The strains shared high similarity based on PFGE, being clustered into two groups; however, nine PFGE patterns were observed. The *hlyA* positive isolates clustered together in spite of being from different sources. Similarly, isolates positive for either *eae* or *bfp* were placed in the same cluster. The isolates that harbored only the *stx2* virulence gene comprised the biggest cluster. The remaining cluster was made up of isolates that harbored only the *stx1* virulence gene and were all isolated from goats. Our findings support that the clustered pathotypes of *E. coli* are closely related; however, further investigations with larger numbers of strains, including strains from clinically ill animals, may provide detailed insights for deriving more relevant epidemiological conclusions.

Heatmap analysis grouped isolates from different hosts by phenotypic and genotypic features. Biofilm development, cefpodoxime resistance, cefotaxime resistance, and stx2 gene presence were positively correlated (Spearman's coefficient >0.92; *p* < 0.05). Ceftazidime resistance was associated with ESBL generation and cefotaxime resistance. ESBL production and the *hlyA* gene were likewise positively correlated. However, a previous study from Iran reported a negative correlation between the presence of the *hlyA* gene and ESBL production [41]. In this study, no significant statistical association was observed between pathotypes and biofilm production. This is in agreement with a previous observation [42].

One limitation of the present attempt is that, due to limited resources, we were unable to investigate faecal samples from human contacts or animal owners to establish the zoonotic transmission potential of the isolated pathogens. Therefore, there is a need to undertake large-scale studies, including samples from animals, the environment, and tribal populations rearing animals, to elucidate the definite public health risk posed by the pathotypes of *E. coli.* Additionally, whole genome-based analysis of pathogenic *E. coli* strains from multiple sources is also required to study the real epidemiological link and impact.

## 5 Conclusions

The findings of the current study reveal that food animals raised by tribal communities and smallholder farmers in Northeastern India can serve as a reservoir of highly virulent biofilm-forming pathotypes of *E. coli*, specifically STEC/EPEC. A significant majority (92.3%) of the isolated STEC and EPEC strains were identified as biofilm formers, while 46.1% of the isolates exhibited ESBL production. Moreover, the application of PFGE fingerprinting demonstrated the circulation of identical clones among various livestock species and geographically distant sampling locations. STEC and EPEC strains are known to cause fatal and difficult-to-treat diseases in humans. Therefore, monitoring these pathogens in food animals is essential for optimizing public health through a one health strategy. Controlling STEC and EPEC in food animals ensures safer animal-derived food for consumers.

## Funding statement

No specific fund was obtained to undertake this study.

## Author contribution statement

A. Arun Prince Milton: Conceived and designed the experiments, performed the experiments, analyzed and interpreted the data and wrote the paper.

K. Srinivas: Performed the experiments and analyzed and interpreted the data.

Vanita Lyngdoh: Performed the experiments.

Aleimo G. Momin: Performed the experiments.

Naphisabet Lapang: Performed the experiments.

G. Bhuvana Priya: Analyzed and interpreted the data and wrote the paper.

Sandeep Ghatak: Analyzed and interpreted the data R.K. Sanjukta: Analyzed and interpreted the data.

Arnab Sen: Contributed reagents, materials and analysis tools.

Samir Das: Contributed reagents, materials and analysis tools and wrote the paper.

## Data availability statement

Not Applicable.

## Declaration of competing interest

The authors declare that they have no known competing financial interests or personal relationships that could have appeared to influence the work reported in this paper.
